# Expanding conservation culturomics and iEcology from terrestrial to aquatic realms

**DOI:** 10.1371/journal.pbio.3000935

**Published:** 2020-10-29

**Authors:** Ivan Jarić, Uri Roll, Robert Arlinghaus, Jonathan Belmaker, Yan Chen, Victor China, Karel Douda, Franz Essl, Sonja C. Jähnig, Jonathan M. Jeschke, Gregor Kalinkat, Lukáš Kalous, Richard Ladle, Robert J. Lennox, Rui Rosa, Valerio Sbragaglia, Kate Sherren, Marek Šmejkal, Andrea Soriano-Redondo, Allan T. Souza, Christian Wolter, Ricardo A. Correia

**Affiliations:** 1 Biology Centre of the Czech Academy of Sciences, Institute of Hydrobiology, České Budějovice, Czech Republic; 2 University of South Bohemia, Faculty of Science, Department of Ecosystem Biology, České Budějovice, Czech Republic; 3 Mitrani Department of Desert Ecology, The Jacob Blaustein Institutes for Desert Research, Ben-Gurion University of the Negev, Midreshet Ben-Gurion, Israel; 4 Leibniz-Institute of Freshwater Ecology and Inland Fisheries, Berlin, Germany; 5 Division of Integrative Fisheries Management, Faculty of Life Sciences, Humboldt-Universität zu Berlin, Berlin, Germany; 6 School of Zoology, Tel-Aviv University, Tel-Aviv, Israel; 7 Steinhardt Museum of Natural History, Tel-Aviv University, Tel-Aviv, Israel; 8 School for Resource and Environmental Studies, Dalhousie University, Halifax, Canada; 9 The Jacob Blaustein Center for Scientific Cooperation, The Jacob Blaustein Institutes for Desert Research, Ben-Gurion University of the Negev, Midreshet Ben-Gurion, Israel; 10 Department of Zoology and Fisheries, FAFNR, Czech University of Life Sciences Prague, Prague, Czech Republic; 11 Department of Botany and Biodiversity Research, University of Vienna, Vienna, Austria; 12 Geography Department, Faculty of Mathematics and Natural Sciences, Humboldt-Universität zu Berlin, Berlin, Germany; 13 Institute of Biology, Freie Universität Berlin, Berlin, Germany; 14 Berlin-Brandenburg Institute of Advanced Biodiversity Research, Berlin, Germany; 15 Institute of Biological and Health Sciences, Federal University of Alagoas, Av. Lourival Melo Mota, Maceió, Brazil; 16 Laboratory for Freshwater Ecology and Inland Fisheries at NORCE Norwegian Research Center, Bergen, Norway; 17 MARE—Marine and Environmental Sciences Centre, Laboratório Marítimo da Guia, Faculdade de Ciências da Universidade de Lisboa, Cascais, Portugal; 18 Institut de Ciències del Mar, CSIC, Barcelona, Spain; 19 CIBIO/InBio, Centro de Investigação em Biodiversidade e Recursos Genéticos, Laboratório Associado, Universidade do Porto, Vairão, Portugal; 20 CIBIO/InBio, Centro de Investigação em Biodiversidade e Recursos Genéticos, Laboratório Associado, Instituto Superior de Agronomia, Universidade de Lisboa, Lisbon, Portugal; 21 Helsinki Lab of Interdisciplinary Conservation Science, Department of Geosciences and Geography, University of Helsinki, Helsinki, Finland; 22 Helsinki Institute of Sustainability Science, University of Helsinki, Helsinki, Finland; 23 DBIO & CESAM-Centre for Environmental and Marine Studies, University of Aveiro, Aveiro, Portugal

## Abstract

The ongoing digital revolution in the age of big data is opening new research opportunities. Culturomics and iEcology, two emerging research areas based on the analysis of online data resources, can provide novel scientific insights and inform conservation and management efforts. To date, culturomics and iEcology have been applied primarily in the terrestrial realm. Here, we advocate for expanding such applications to the aquatic realm by providing a brief overview of these new approaches and outlining key areas in which culturomics and iEcology are likely to have the highest impact, including the management of protected areas; fisheries; flagship species identification; detection and distribution of threatened, rare, and alien species; assessment of ecosystem status and anthropogenic impacts; and social impact assessment. When deployed in the right context with awareness of potential biases, culturomics and iEcology are ripe for rapid development as low-cost research approaches based on data available from digital sources, with increasingly diverse applications for aquatic ecosystems.

## Introduction

The digital revolution provides unique opportunities to gain additional or complementary knowledge on the environment and related human values, attitudes, norms, preferences, and behaviors. Culturomics and iEcology are emerging research fields that mine digital data generated by people as part of their daily lives to develop new insights with low sampling costs and high spatiotemporal breadth (S1 and S2 Figs) [1,2]. The methods of culturomics, which focuses on the study of human culture through the quantitative analysis of large bodies of digital data [3], are being used to study contemporary problems in conservation [1] through the prism of human–nature interactions. Such applications include the study of societal interest in different organisms and ecosystem services; attitudes of stakeholders and the general public towards environmental impacts of development; human behavior concerning ongoing management and conservation efforts; and the distribution, intensity, and spatiotemporal dynamics of anthropogenic threats and resource uses [4–13].

iEcology studies ecological patterns and processes using data generated for other purposes and stored digitally [2]. It uses similar data sources and analytical tools as culturomics but extracts information that addresses broad ecological questions such as species occurrences, distributional range shifts, population dynamics, life history, ecological status, and monitoring of target taxa such as alien, rare, or threatened species (S1 Table) [14,15].

Scientists working on the terrestrial realm have harnessed the potential of culturomics and iEcology applications, but their use in aquatic realms is far more limited and faces greater challenges. Here, we advocate for a wider application of these new digital approaches to the science and conservation of freshwater and marine environments and those who depend on them, discuss the relevance and potential of such applications, present associated challenges and limitations, and highlight key areas in which these new approaches may have the most impact.

### Making a case for aquatic culturomics and iEcology

The aquatic environment comprises both marine and freshwater ecosystems, which together cover approximately 72% of the Earth's surface. These ecosystems provide essential services to people, with the majority of human populations living along coasts and within river basins, and thus are widely recognized as conservation priorities [16–18]. Indeed, freshwater and marine coastal habitats are severely threatened by the synergistic effects of anthropogenic pressures such as habitat loss, damming, invasive alien species, water extraction, pollution, and unsustainable harvest [19–23]. As a result, aquatic species face disproportionately higher extinction risks than terrestrial species [24,25], which impacts the well-being of communities that depend on aquatic ecosystems.

Current levels of research, monitoring, and action remain insufficient to cope with the impacts that aquatic habitats face and their consequent effects on people. Research in aquatic environments is hindered by limited accessibility and low species detectability [26,27]. Importantly, environmental impacts in aquatic environments frequently occur faster than they can be actively monitored and understood. Furthermore, conducting high-quality social science research to understand human values, attitudes, behaviors, and knowledge towards aquatic environments is time-consuming and often costly while also lagging behind fast ecological changes or happening at scales that do not match local ecological change [28,29].

Culturomics and iEcology can provide valuable contributions to aquatic sciences and conservation as both complementary and unique sources of information. The chronic data and research deficits of aquatic systems [17,30] call for the development of novel research methods. Because culturomics and iEcology take advantage of available data, they are also far less costly than field sampling and social surveys.

Yet applying culturomics and iEcology in aquatic environments faces challenges. Online data sources dedicated to aquatic environments tend to be considerably more limited compared to terrestrial sites. Moreover, human–nature interactions are especially difficult to document in these ecosystems with digital technology (often requiring more specialized equipment—for example, waterproof cameras) and have uneven spatial coverage, with scarce data from areas farther from the shore and underwater. Nevertheless, these approaches still represent a rich source of information, and their potential should improve as technological advances such as underwater wireless connection provide new opportunities to document our interactions underwater [31].

Below, we present promising areas of application of culturomics and iEcology, focusing on those that are particularly relevant for aquatic ecosystems, including the detection and distribution of threatened, rare, and alien species; ecosystem status and anthropogenic impacts; wildlife and fisheries management; flagship species identification; protected areas management; and social impact assessment for development proposals. We further provide examples from published works (S1 Table).

### Detection, mapping, and monitoring of threatened, rare, and alien species

Compared to their terrestrial counterparts, many aquatic species are chronically undersampled. Since marine and freshwater surveys are comparatively expensive, harnessing alternative sources of data on species distributions is critical. One of the most common ecological applications of online digital data is to explore species occurrences and distribution [2]. The ever-expanding stream of user-generated content (including geospatially coded photographs, videos, and audio recordings) in online platforms such as Facebook, Instagram, YouTube, or news media can be used to identify and detect species presence and map their distributions, population densities, and group sizes to monitor their spatiotemporal dynamics. Such sources could be particularly relevant for identifying new or remnant populations of rare or threatened species, as well as for early detection and monitoring of alien species. Recordings can also provide data on both species' presences and absences. Such methods have so far been applied to monitor various aquatic mammals, including cetaceans in the Mediterranean Sea [32,33], Hawaiian monk seals (*Neomonachus schauinslandi*) in the Hawaiian Islands [34], and Eurasian otters (*Lutra lutra*) in South Korea [35]. Online media can also be used to study spatiotemporal intraspecific phenotypic variation [36], as well as species co-occurrence patterns. The imminent arrival of automatic species identification following progress in machine learning methods [37–39] and growing taxonomic reference image databases [40] will further increase the utility of such approaches. The application of marine and freshwater ecoacoustics (i.e., the study of soundscapes and the relationship between sound and the environment) [41,42] to video and audio documents made available online could also further enhance these capabilities. While most such recordings would not be useful as data sources, the sheer number of videos uploaded daily will ensure that even a very small proportion of usable documents will result in large data sets suitable for analysis. Soundscape assessment approaches have been already demonstrated in the terrestrial realm [43]. Digital sources could also be mined for past occurrences as well as used for monitoring species in real time. Nevertheless, the availability of spatial digital data is strongly driven by species characteristics, including charismatic traits, body size, conspicuousness, proximity to humans, and socioeconomic value, restricting most studies to vertebrates. One approach that could greatly facilitate monitoring of noncharismatic and less conspicuous elements of biodiversity would be the development of automated species recognition to analyze background information in digital data, such as species captured unintentionally in the background of photos and videos. Such monitoring methods could also prove more comprehensive than monitoring based on targeted videos and images (i.e., in which the species was filmed intentionally) since they entail fewer potential biases due to human agency (see below) and might be especially relevant for monitoring sessile species, e.g., vegetation [44].

### Ecosystem status and impacts

Digital approaches can complement conventional methods to detect and monitor changes in ecological community and population structure, phenology, and impacts of extreme events and climate change [15,44,45]. They can also be used as early warning systems for ecosystem phase shifts or emergent impacts [46]. For example, analyzing Google Images revealed a likely climate-change–driven phenological shift in the breeding periods of Japanese dace (*Triblodon hakonensis*) [47] and identified the prevalence and intensity of parasite-induced coloration phenomenon in Caribbean reef fish [48]. Digital photo analysis was also used to identify the extent of anthropogenic impacts and degradation of coral reefs from central Pacific atolls and the Caribbean Sea [4]. iEcology cannot replace standard field studies, but its broad geographical reach could make it an effective tool for preliminary screening and identification of priority areas to focus research effort.

### Wildlife and fisheries management

**Fig 1:**
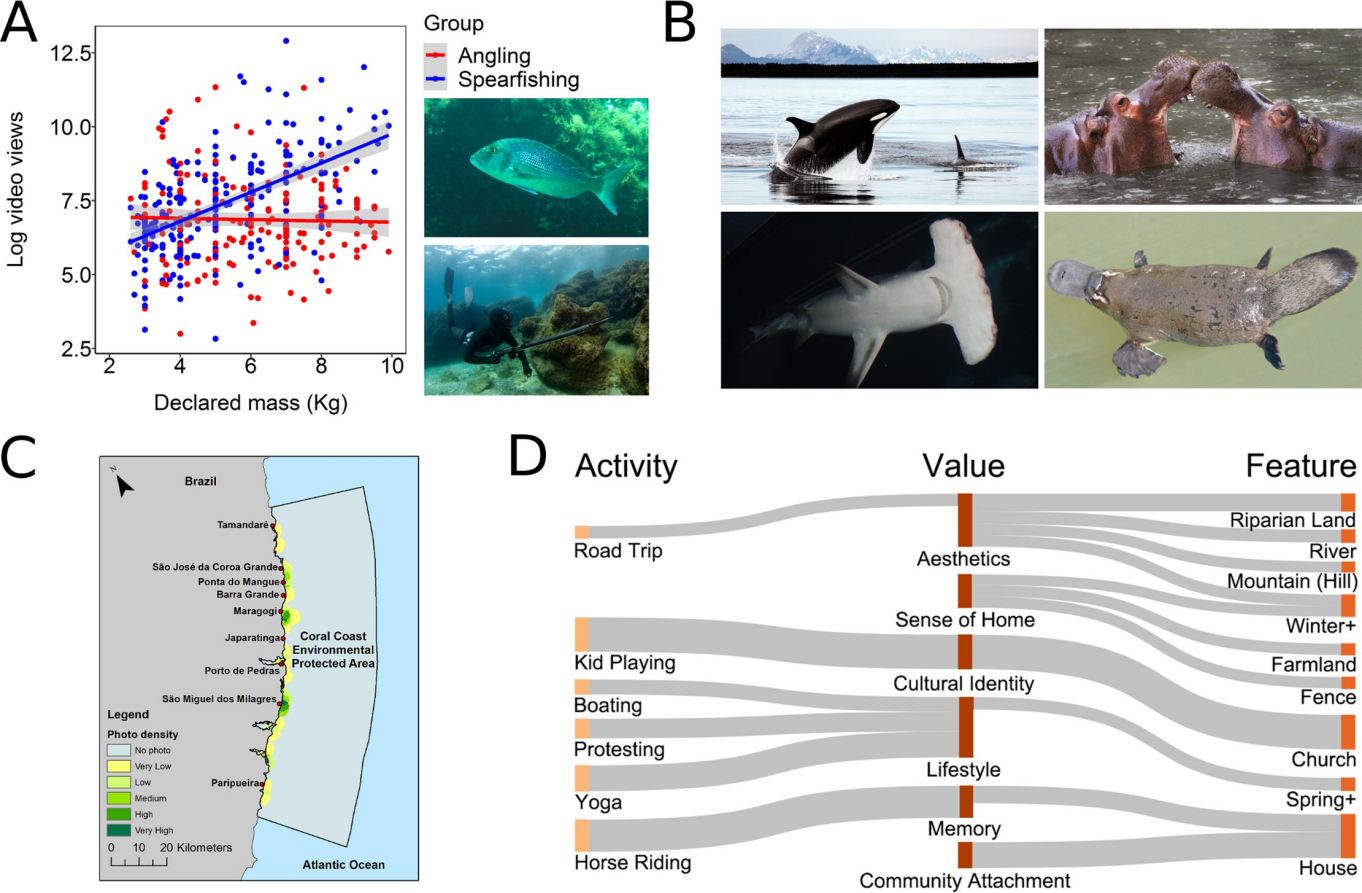
Examples of aquatic culturomics and iEcology studies. (A) Social engagement of marine recreational anglers and spearfishers targeting common dentex (*Dentex dentex*), an iconic species for Mediterranean fisheries, based on videos posted on YouTube [12]; upper photo—common dentex, lower photo—spearfisher. (B) Potential aquatic flagship species identified based on their popularity (relative internet search frequency) [59]; presented are top-ranked marine (killer whale, *Orcinus orca*, and great hammerhead, *Sphyrna mokarran*) and freshwater species (hippopotamus, *Hippopotamus amphibius*, and platypus, *Ornithorhynchus anatinus*). (C) Mapping of cultural ecosystem service hotspots in a marine protected area, based on social media photographs [11]. (D) Conceptual landscape perception map, based on statistical relationships between activities, values, and features coded from landscape images and captions on Instagram, from the proposed headpond area of the now-approved Site C dam, Peace River, British Columbia, Canada [6,68]. See the supporting information (S1 Text) for image attributions.

Culturomics and iEcology can help wildlife and fisheries managers monitor distributions, compositions, and dynamics of communities; fishing or hunting practices; fishers' or hunters' activities and behaviors; fisheries or managed harvest sustainability; and wildlife trade. Several studies have inferred fish population trends and overfishing from temporal trends in fish size and composition using digitally stored data such as photographs and news articles [14,49,50]. Furthermore, historical data from digitized texts, photographs, or ship logs can provide insights into the distribution or abundance of species at times when scientific sampling data are limited [51]. Culturomics can shed light on the behavior and preferences of fishers [52], for example, by assessing regional characteristics of recreational fisheries based on YouTube videos or discussion forums (Fig 1A) [7,9,12] or by analyzing internet search frequency to explore global trends in fishing interest and seasonality [5]. These approaches can also be used to monitor the effectiveness of fisheries management initiatives by tracking attitudes and compliance of fishers before, during, and after implementation of regulations and to anticipate reactions.

### Flagship umbrella species

Flagship and umbrella species (or the integration of both concepts as “flagship umbrella species”) [53], respectively, represent conservation surrogate species with a potential to be used as the focus of a broader conservation marketing campaign [54] and species whose conservation confers a protective umbrella to numerous co-occurring species [55]. Both concepts are still underutilized in aquatic environments (with the exception of some marine mammals and sea turtles) [56,57] because of the lower accessibility and visibility of aquatic species and ecosystems. Culturomics and iEcology can be valuable approaches to identify flagship and umbrella species and monitor their public uptake [1]. Culturomics can help identify promising flagship species based on societal interest across many candidate species (Fig 1B) [58,59], while iEcology can help identify potential umbrella species by mapping their distribution and overlaps with key habitats and co-occurring species. Culturomics can also help gauge the effects of public awareness campaigns and behavior change interventions that employ flagship species [60] and help assess and adapt social marketing strategies. Examples in which these concepts have been already applied in the aquatic realm show that they can work well in practice. For example, species such as salmonids, sturgeons, and freshwater dolphins have been promoted as freshwater flagships by the International Union for Conservation of Nature (IUCN), World Wide Fund for Nature (WWF), and International Commission for the Protection of the Danube River (ICPDR) [57].

### Management of protected areas and landscapes

Culturomics can provide valuable and cost-effective information for managing protected areas, including data on tourism pressures, use of different habitats for recreation, cultural ecosystem services, and societal awareness, attitudes, and sentiments [61,62]. Obtained insights can be critical for developing management and marketing programs, especially in protected areas that have no monitoring systems in place [61]. Image-sharing platforms such as Flickr and Instagram provide information on tourist preferences for nature-based experiences in protected areas [63]. Many protected areas are exposed to high tourist visitation frequency, which makes them especially suitable for developing monitoring programs based on culturomics approaches [61,64]. These have been used to assess cultural ecosystem services, as well as tourism preferences and intensity, in Ramsar wetlands in South Korea [10] and India [65] and in marine protected areas such as Brazil's Costa dos Corais (Fig 1C) [11] or Australia's Great Barrier Reef [66]. Such studies mostly map geocoded images, which can be augmented with automated facial expression analyses or sentiment analyses of captions [10,11,13,65,66] to provide better insight into tourist attitudes and preferences.

### Social impact assessment

Social media and other digital data sources can be effectively utilized to assess the social impacts of construction or infrastructure development [6], to evaluate activities and values associated with landscape features and cultural ecosystem services, and to help anticipate the expected impacts of planned projects [67,68]. Such projects include hydroelectric dams, offshore wind parks, oil platforms, gravel extraction, channelization, embankments, and development of marinas, ports, and touristic resorts. These approaches have already been used for social impact assessment of planned and existing hydroelectric dams on the Peace and St. John Rivers in Canada using Instagram data (Fig 1D) [67,68], as well as the Three Gorges Dam in China using sentiment analysis of news articles, forums, and blogs [69]. While they are yet to be incorporated in environmental decision-making, these methods have a great potential to become relevant part of this process, especially for large-scale projects and impacts, for which traditional social impact assessment methods may be impractical.

### Caveats and challenges in culturomics and iEcology in aquatic environments

**Fig 2:**
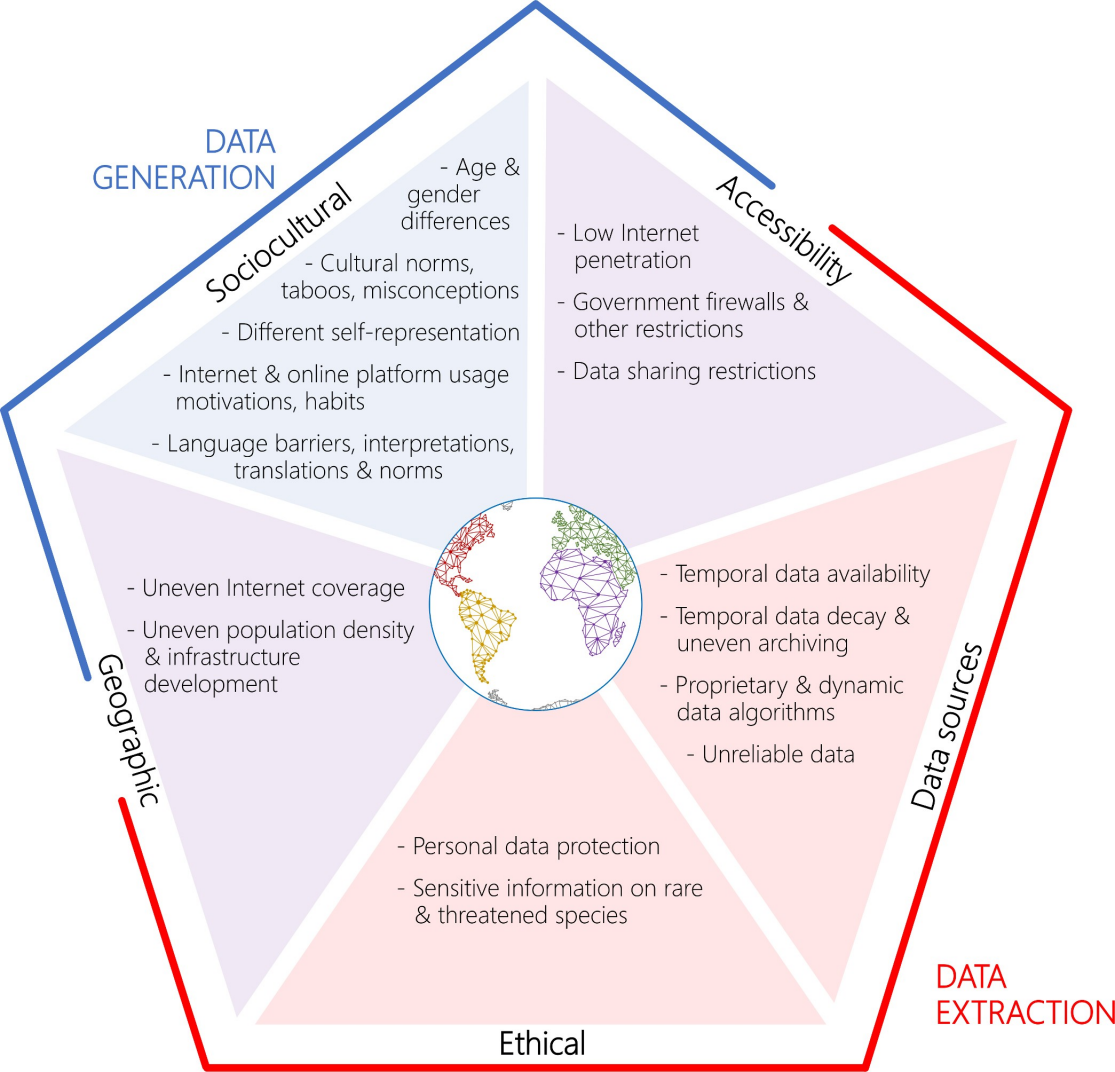
Overview of challenges and biases associated with conservation culturomics and iEcology research, divided into 5 groups: sociocultural aspects, accessibility issues, geographic factors, issues related to data sources, and ethical issues. It should be noted that some of the listed issues also represent key research subjects for the field of culturomics.

Ensuring reliable results when using digital data for the purposes described here faces important caveats and challenges. These issues are linked to both data generation and data extraction and encompass sociocultural aspects, accessibility, geographic factors, data sources, systematic differences between users and nonusers of digital data, and ethical considerations (Fig 2).

Digital data availability and representativeness can be affected by various cultural, political, and socioeconomic factors, as well as demographic characteristics such as age, gender, and education. Furthermore, biases may also arise from different cultural norms, taboos, and misconceptions, as well as differences in internet and online platform usage motivations and habits, and their changes over time [70,71]. Social media users often represent a specific stratum of the population, and data may be biased towards more active users and specific social groups [61,71]. For example, recreational fishers posting about their catch and expressing their opinion may deviate from a random sample [72], and their featured species may be biased towards larger and more impressive species and individuals [12]. Furthermore, rural, traditional, and indigenous societies are usually underrepresented in digital data, and data generated by tourism can interfere with assessments of local population attitudes and behaviors [1]. Another prevalent challenge is that the way people represent themselves on social media is often far removed from reality, and their interactions with others are filtered to make their representation appropriate to the intended audiences [61,73]. Digital data are also characterized by a range of linguistic challenges, including language barriers, semantic complexity, linguistic diversity and instability, and challenges related to interpretations, translations, and language norms [1,70,74].

The highly uneven spatial coverage of the internet and its users is exacerbated in aquatic realms. Digital data are also much sparser, with coverage tending to decrease with distance from shore and water depth and concentrating along transportation routes, in areas with higher population density, and in recreation areas.

Digital data are also limited temporally. While there are increasing efforts to digitize past content, in many cases, the earliest available data are limited. Furthermore, data access and use are hindered by nontransparent and dynamic data-access algorithms (such as Application Programming Interfaces [APIs]), often with limited access because of proprietary constraints, data sharing restrictions and firewalls, and limited replicability [1,61,70,75]. Online data are also characterized by temporal decay (i.e., webpage removal, data loss and deletion) and uneven archiving, as well as by the presence of unreliable data (i.e., incorrect spatial and temporal information, nonexpert species identifications, false information, etc.).

The fields of culturomics and iEcology are still developing established frameworks and protocols of good practice to tackle privacy issues and ethical use [71,76]. Publicly available digital data, especially those shared on social media, often involve sensitive personal information that requires establishing a set of guidelines to ensure ethical web-scraping practices [76]. Furthermore, digital data can reveal sensitive information on rare and threatened species, such as precise locations and other attributes that could facilitate poaching and unsustainable harvesting [2].

In general, digital data are nonrandom in extent and depth and vary among users, regions, cultures, time frames, and taxonomic groups [2,75] and require calibration and validation to quantify such biases. Digital data should therefore be used with due caution in the right study context while controlling for biases. For example, extrapolating compliance or user perspectives on an issue expressed online will unlikely scale to the entire population without correcting for sample bias. Nonetheless, questions related to local communities and particular societal groups can be addressed without making population-level inferences. Some of the biases related to digital data should diminish over time as internet penetration improves. Inferences obtained from digital data can be made more robust by simultaneous use and cross-validation of multiple digital data sources such as different search engines, social media platforms, online news, and digital encyclopedias [70,77]. Additionally, whenever possible, digital data should be validated through ground-truthing and triangulation with other data sources such as systematic surveys, remote sensing, and citizen science [2,78]. Finally, culturomics and iEcology methods may be also useful to identify new problems, patterns, and hypotheses for more conventional studies in which biases can be better controlled.

## Conclusions

Culturomics, iEcology, and other emerging digital approaches have great potential to produce novel and valuable insights into the sustainable management and conservation of ecosystems and strengthen ongoing research efforts. We demonstrated the potential of these new approaches and advocated for expanding it into aquatic realms, where they are likely to increase quickly as new tools are developed and their limitations and biases are better understood and addressed (Fig 2). Emerging technologies such as automated web crawling and data processing, machine learning, automatic species identification, apps, and ecoacoustics could further enhance their utility and uptake by the scientific and conservation communities [2]. Ultimately, we envision the potential for a global digital observatory of Earth, an online platform established for continuous collection and processing of key digital data from a wide variety of sources that could provide near real-time information on ecosystem change and human–nature interactions.

With the right tools and expertise, digital data represent a rich and unique resource for both aquatic and terrestrial research. They can also contribute to monitoring progress towards the Sustainable Development Goals (SDGs) and the Post-2020 Biodiversity Goals of the Convention on Biological Diversity (CBD) [79]. For example, they can contribute to improved knowledge and the development of research capacities in aquatic research (SDG target 14.A) and can support ongoing research and monitoring efforts related to the management of aquatic ecosystems (SDG 14.2, 14.5 and 15.1, CBD #1 and 2), biological invasions (SDG 15.8, CBD #3), climate change (SDG 13.3, CBD #6), wildlife and fishery management and trade (SDG 14.4 and 15.7, CBD #5), biodiversity protection (SDG 15.5, CBD #18), and sustainable tourism (SDG 12.B). Moreover, they can also support efforts towards human-oriented SDGs, such as those related to the impacts of poor source water quality (SDG 6) [80]. We call upon the scientific community to explore and engage with culturomics and iEcology approaches as well as to actively seek collaborations across disciplines, especially with computer and social scientists, to provide opportunities for the most effective and innovative transdisciplinary analyses of the pressing issues in the conservation of biodiversity [2,81,82].

## Supporting information

S1 Fig Conceptual diagram with key differences among culturomics, iEcology, and other related approaches such as citizen science and social surveys. Differences are based on the object of study (human–nature interactions or nature itself) and the type of data generation (passive or active). Data sets generated by citizen science, social surveys, and other approaches can also represent data sources for iEcology and culturomics, as indicated by arrows. Drawings illustrate some applications of culturomics and iEcology for aquatic research: 1) fisheries management; 2) social impact assessment; 3) detection, mapping, and monitoring of threatened, rare, and alien species; 4) ecosystem status and anthropogenic impacts; and 5) identification of aquatic flagship and umbrella species.
(TIF)

S2 Fig Conceptual workflow of aquatic culturomics and iEcology research. The figure highlights how data are obtained, processed, and analyzed to gain insights on aquatic ecosystems using iEcology or culturomics approaches. The shaded-out region on the left of the figure represents the more traditional aquatic research avenues that lie outside the scope of this manuscript. However, once their underlying data are digitized and shared, they too can contribute to iEcology and culturomics explorations.
(TIF)

S1 Table Applications of culturomics and iEcology in aquatic research. A compilation of available examples of culturomics and iEcology studies applied in aquatic research, with associated information on studied regions and countries, data sources used, and research topics.
(XLS)

S1 Text Image attributions for Fig 1. (DOC)

## Abbreviations

API: Application Programming Interface
CBD: Convention on Biological Diversity
ICPDR: International Commission for the Protection of the Danube River
IUCN: International Union for Conservation of Nature
SDG: Sustainable Developmental Goal
WWF: World Wide Fund for Nature
