# Supplementary figures and images for: Expanding conservation culturomics and iEcology from terrestrial to aquatic realms
Source: PLoS Biol. 2020 Oct 29;18(10):e3000935. doi: 10.1371/journal.pbio.3000935 (PMC7595319; doi:10.1371/journal.pbio.3000935)

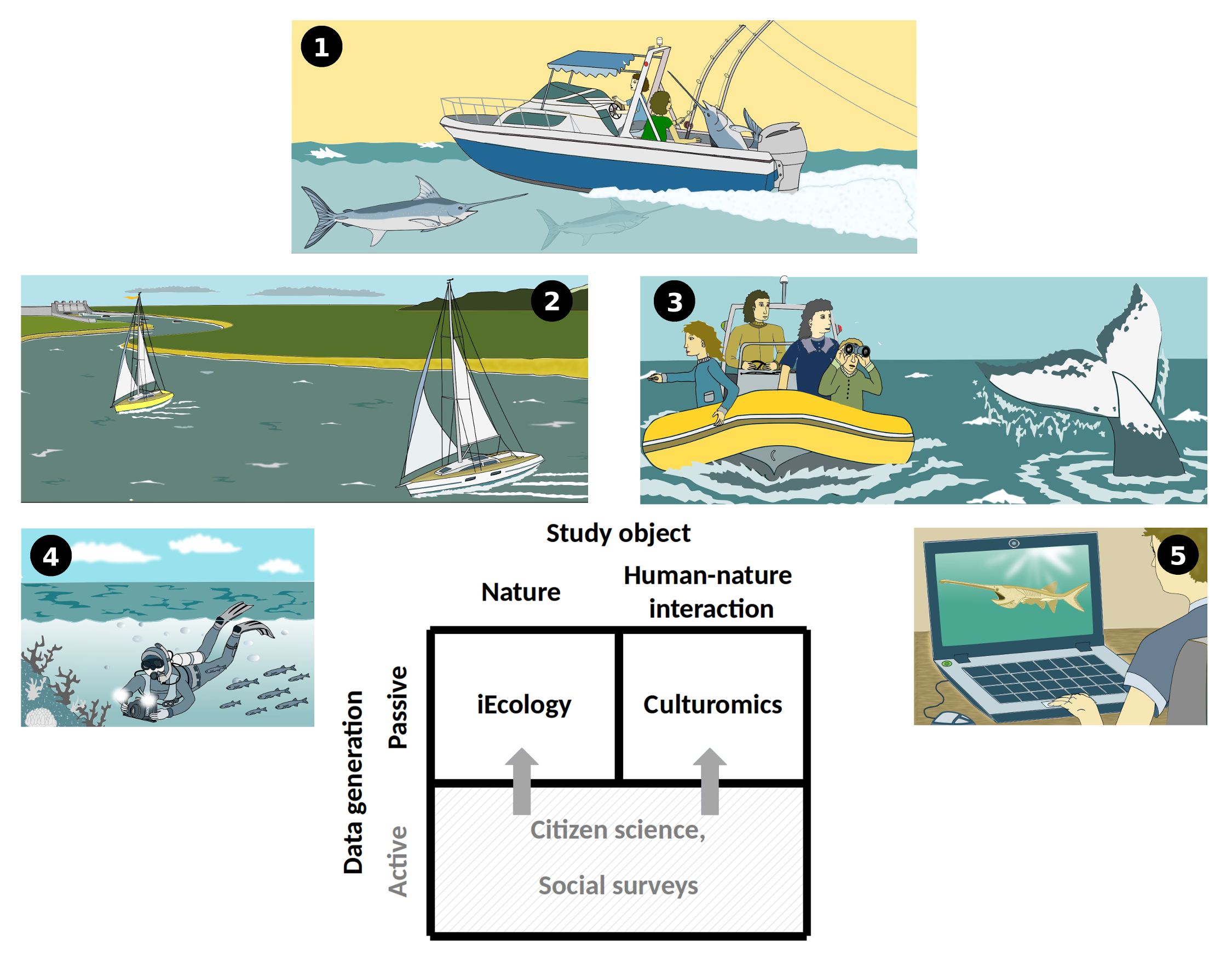

Supplement: S1 Fig — Differences are based on the object of study (human–nature interactions or nature itself) and the type of data generation (passive or active). Data sets generated by citizen science, social surveys, and other approaches can also represent data sources for iEcology and culturomics, as indicated by arrows. Drawings illustrate some applications of culturomics and iEcology for aquatic research: 1) fisheries management; 2) social impact assessment; 3) detection, mapping, and monitoring of threatened, rare, and alien species; 4) ecosystem status and anthropogenic impacts; and 5) identification of aquatic flagship and umbrella species. (TIF) [file pbio.3000935.s001.tif]

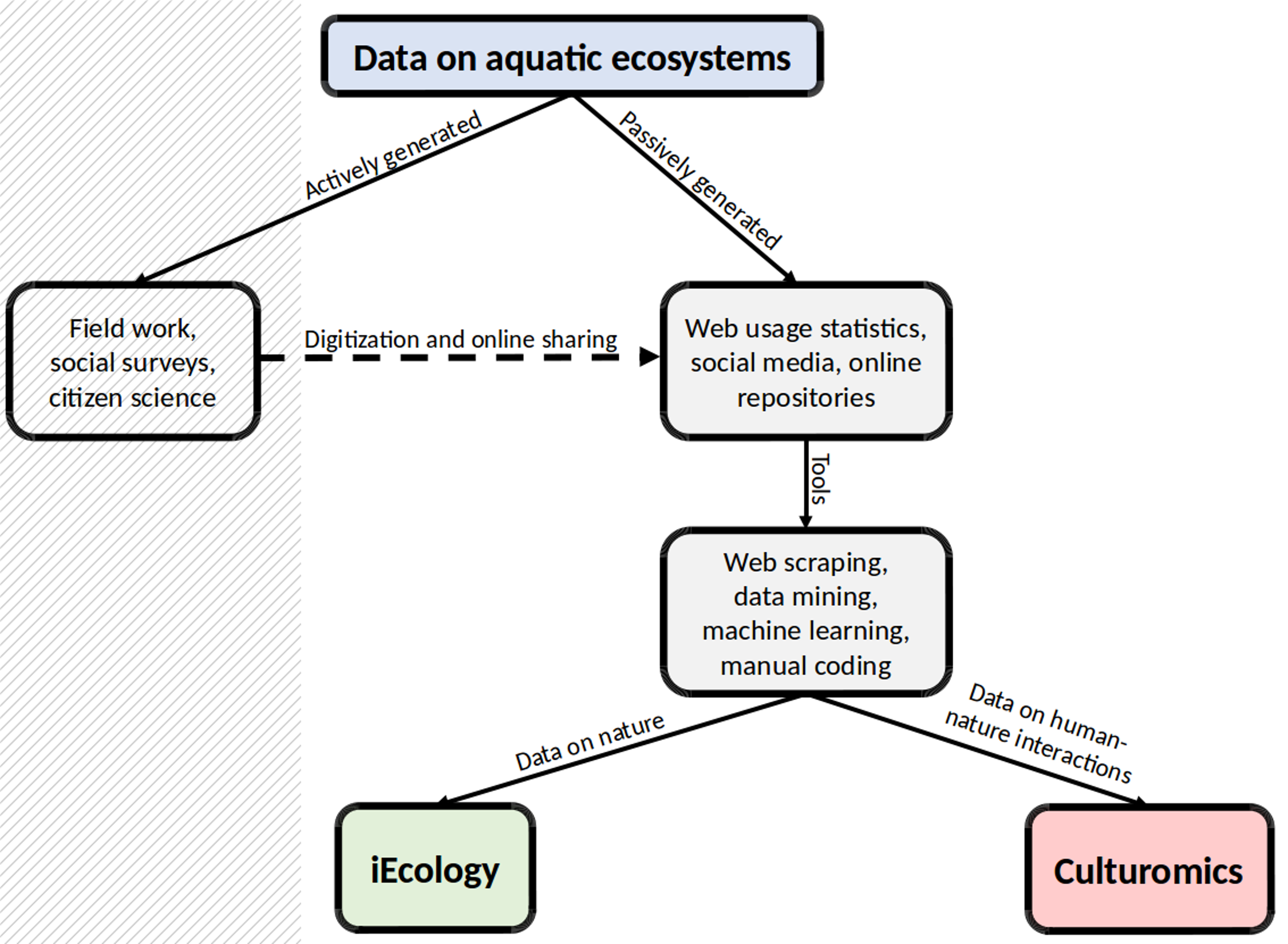

Supplement: S2 Fig — The figure highlights how data are obtained, processed, and analyzed to gain insights on aquatic ecosystems using iEcology or culturomics approaches. The shaded-out region on the left of the figure represents the more traditional aquatic research avenues that lie outside the scope of this manuscript. However, once their underlying data are digitized and shared, they too can contribute to iEcology and culturomics explorations. (TIF) [file pbio.3000935.s002.tif]
