# Supplementary material for: Expanding conservation culturomics and iEcology from terrestrial to aquatic realms
Source: PLoS Biol. 2020 Oct 29;18(10):e3000935. doi: 10.1371/journal.pbio.3000935 (PMC7595319; doi:10.1371/journal.pbio.3000935)
Supplement: S1 Text — (DOC) [file pbio.3000935.s004.doc]

**Supplementary Material**

**Expanding conservation culturomics and iEcology from terrestrial to aquatic realms**

Ivan Jarić, Uri Roll, Robert Arlinghaus, Jonathan Belmaker, Yan Chen, Victor China, Karel Douda, Franz Essl, Sonja C. Jähnig, Jonathan M. Jeschke, Gregor Kalinkat, Lukáš Kalous, Richard Ladle, Robert J. Lennox, Rui Rosa, Valerio Sbragaglia, Kate Sherren, Marek Šmejkal, Andrea Soriano-Redondo, Allan T. Souza, Christian Wolter and Ricardo A. Correia

**S1 Text. Image attributions for Fig 1:**

A, upper photo) Common dentex (*Dentex dentex*). Credit: David Mandos

A, lower photo) Spearfisher. Credit: David Mandos

B, upper left photo) Killer whale (*Orcinus orca*). Credit: Christopher Michel

https://commons.wikimedia.org/wiki/File:Orcas_in_Alaska.jpg

Creative Commons Attribution 2.0 Generic license.

B, lower left photo) Great hammerhead (*Sphyrna mokarran*). Credit: Jim Capaldi

https://commons.wikimedia.org/wiki/File:Sphyrna_mokarran_camden.jpg

Creative Commons Attribution 2.0 Generic license.

B, upper right photo) Hippopotamus (*Hippopotamus amphibius*). Credit: William Warby

https://commons.wikimedia.org/wiki/File:Hippopotamus_amphibius_Whipsnade_Zoo.jpg

Creative Commons Attribution 2.0 Generic license.

B, lower right photo) Platypus (*Ornithorhynchus anatinus*). Credit: Brisbane City Council

https://commons.wikimedia.org/wiki/File:Feeding_Platypus_(6811147158).jpg

Creative Commons Attribution 2.0 Generic license.
